# Second Malignant Neoplasms Following Radiotherapy

**DOI:** 10.3390/ijerph9124744

**Published:** 2012-12-18

**Authors:** Sanath Kumar

**Affiliations:** Department of Radiation Oncology, Henry Ford Hospital, Detroit MI 48202, USA; E-Mail: skumar4@hfhs.org; Tel.: +1-313-916-1021; Fax: +1-313-916-3264

**Keywords:** radiation, second malignancy, cancer therapy, side effects, radiotherapy, neoplasm

## Abstract

More than half of all cancer patients receive radiotherapy as a part of their treatment. With the increasing number of long-term cancer survivors, there is a growing concern about the risk of radiation induced second malignant neoplasm [SMN]. This risk appears to be highest for survivors of childhood cancers. The exact mechanism and dose-response relationship for radiation induced malignancy is not well understood, however, there have been growing efforts to develop strategies for the prevention and mitigation of radiation induced cancers. This review article focuses on the incidence, etiology, and risk factors for SMN in various organs after radiotherapy.

## 1. Introduction

Radiotherapy is an integral part of cancer management, with more than 50% of all the patients undergoing radiation treatment [1]. With recent advances in surgical techniques, chemotherapy, and radiotherapy, there has been a significant increase in the number of long-term cancer survivors [2]. Although this is great news in the fight against cancer, many patients treated with radiation therapy suffer from adverse effects, including the development of a second malignant neoplasm (SMN) [3,4]. There is a growing concern about SMN, as it may lead to a decrease in the overall survival after the treatment of primary cancers, especially in pediatric patients.

Although radiation exposure is a well-established risk factor for developing SMN, estimating the true incidence of radiation-induced SMN is difficult. This is due to the fact that, in addition to radiation exposure, the genetic abnormalities (e.g., Li-Fraumeni syndrome) and risk factors associated with primary tumors (e.g., smoking) could predispose the individuals to develop a second cancer [5,6]. Also, the risk estimation of radiation induced SMN in various organs is complicated by the fact that no appropriate control group exists, except for a few sites like the prostate and cervix, where surgery could be used as an alternative to radiotherapy. More importantly, modern treatment techniques allow for conformal radiation delivery and accurate estimation of radiation dose in the treatment field, both of which were lacking in the past. Hence, the risk estimates based on older studies may not accurately represent modern day practices. In this review, putative etiology and risk factors for radiation-induced SMN will be discussed. This will be followed by brief a summary of SMN following radiation treatment of various cancers.

## 2. Pathogenesis of Radiation Induced SMN

Cancer is a multi-step process which involves multiple genetic changes and results in the transformation of normal cells into malignant cells. These genetic alterations are a result of various environmental and endogenous DNA-damaging agents [7]. Ionizing radiation has long been recognized to have carcinogenic potential. One of the most conclusive pieces of evidence for radiation induced SMN came from the long term follow-up of A-bomb survivors in Hiroshima and Nagasaki, which showed an increase in the incidence of leukemia and solid tumors [8]. Various mechanisms have been proposed for the pathogenesis of radiation-induced SMN [9,10]. Exposure to low dose radiation is known to cause base damage, single strand DNA breaks, and double strand breaks (DSBs). The single strand breaks and base damage occur in “clusters” following radiation exposure, and they can be converted to DSBs during cell replication. The DSBs could lead to gene mutations, which then lead to a malignant transformation of the radiated cell [9]. Also, impairment in the DNA repair proteins, which normally protect against DNA damage, could lead to increased susceptibility to radiation induced SMN. For example, mutations affecting ataxia telangiectasia mutated (ATM), a protein which senses DNA damage and initiates a repair cascade, can lead to increased radiosensitivity and cancer susceptibility [11]. It has been observed that radiation doses of <0.2 Gy fails to activate the G2/M cell cycle check point [12]. This could result in failure to repair DNA damage and could result in carcinogenesis [12]. Another mechanism that has been proposed to account for SMN at sites distant from the primary treatment area is the radiation induced bystander effect and tissue inflammation [13]. Bystander effect involves intercellular communication through gap junctions and systemic cytokine signaling. Radiation induced SMN has also been observed at high doses of radiation of up-to 45 Gy [10]. This phenomenon is thought to be due to the accelerated repopulation of pre-malignant cells during and after radiation, which could potentially counteract the effects of cell killing at higher doses [10].

## 3. Temporal Relationship between Radiation Exposure and Development of SMN

**Figure 1:**
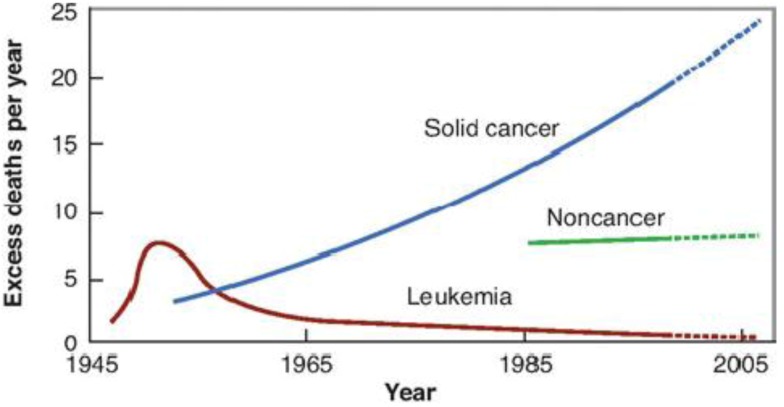
Time course of SMN development following A-bomb explosion in 1945. Leukemia appeared first followed by solid cancers several years later. There was also an excess of non-cancer deaths from stroke and heart disease by the late 1980s (courtesy of Kiyohitko Mabuchi, National Institutes of Health, USA).

Evidence of the long-term effects of radiation, including SMN in humans has been well documented in atomic bomb survivors from Hiroshima and Nagasaki [12,14]. The latent period for the development of leukemia is reported to be in the range of 5–10 years, and about 10–60 years for solid tumors (Figure 1). The development of SMN in patients treated with radiotherapy has been reported to follow a similar timeline [14,15,16,17,18].

## 4. Age

**Figure 2:**
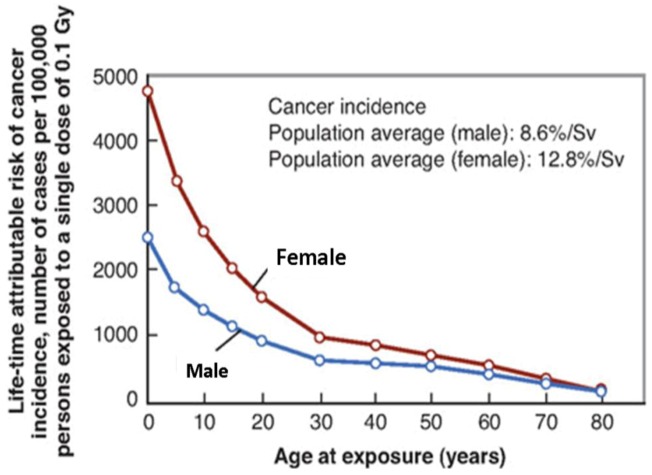
Cancer incidence as a function of age for males and females based on A-bomb data as calculated by BEIR VII committee (reproduced with permission from reference [14]).

Multiple epidemiological studies have confirmed the importance of age in predicting SMN risk at the time radiation exposure. For the same dose, patients exposed to radiation during childhood are at a significantly higher risk for developing SMN compared to those exposed at older age [19,20]. Children are considered to be 10 times more sensitive to radiation induced SMN than are adults [20]. Epidemiological data from Japanese A-bomb survivors reveal a dramatic decrease in the incidence of SMN as a function of age (Figure 2). The cancer risk decreases from about 15%/Sv of whole-body uniform irradiation for children under 10 years of age to about 1%/Sv for adults exposed at over 60 years of age [20]. Also, many cases of childhood cancer have an underlying germline mutation that may increase the susceptibility to radiation induced SMN. For example, the risk of breast cancer after radiotherapy in known to be greater in patients treated for Hodgkin’s disease than Wilms’ tumor [21]. The smaller body size of pediatric patients infers a further potential increase in risk than that of adult patients, as the organs surrounding the treatment site receive larger doses of the scatter radiation compared to an adult [19].

## 5. Radiosensitivity of the Organ

Radiation-induced SMN may occur in organs within the treatment field (high dose region) or distant organs well outside the radiation field (low dose region). These out-of-field cancers are thought to be due to the low dose of scatter radiation reaching the patient [22]. Another possible reason for carcinogenesis is the phenomenon of radiation-induced bystander effect, where cell-cell signaling and tissue inflammation leading to systemic responses could promote carcinogenesis in the organs outside of the radiation field [13]. Most of the cancers in A-bomb survivors were carcinomas of the lung and digestive tract. However, in patients who undergo radiotherapy, there is an increased risk of sarcomas, in addition to carcinomas, in the high dose regions [19]. Table 1 shows the risk coefficients for developing fatal cancer by organ site according to the National Council on Radiation Protection and Measurements (NCRP) report 116. According to this data, colon, lung, and stomach are the most common sites for developing a fatal SMN after radiation exposure [23]. However, the thyroid gland is known to have a low threshold for radiation induced cancer, especially in children. An increase in the incidence of thyroid cancer has been reported after exposure to a mean organ dose as low as 0.05 Gy in children and young adults [24]. Radiation-induced SMNs are rarely seen in the small intestine, and have been attributed to a low apoptosis threshold in intestinal stem cells following a small amount of DNA damage [25].

**Table 1:** Lifetime probability of developing a fatal second cancer by organ site from NCRP report 116 (Reprinted with permission of the National Council on Radiation Protection and Measurements).

Organ	Probability of fatal malignancy (%/Sv)
Stomach	1.10
Colon	0.85
Lung	0.85
Bone marrow	0.50
Bladder	0.30
Esophagus	0.30
Breast	0.20
Liver	0.15
Ovary	0.10
Thyroid	0.08
Skin	0.02
Bone surface	0.05
Remainder of the body	0.50
Total	5.00

## 6. Gender

For a given dose, females have a higher incidence of radiation-induced SMN compared to men (Figure 2) [26,27]. This could be due to the increased risk of breast and thyroid cancers in females, especially after radiation exposure at a younger age [16]. It has also been hypothesized that an increase in the activity of the cytochrome P450 enzyme and the effects of estrogen may promote carcinogenesis in females [28]. A recent report from Taddei *et al.* on the incidence of SMN following proton craniospinal irradiation indicates that the observed sex differences may be attributed to the greater risk coefficients for most organs in females compared to men [29].

## 7. Radiation Dose

**Figure 3:**
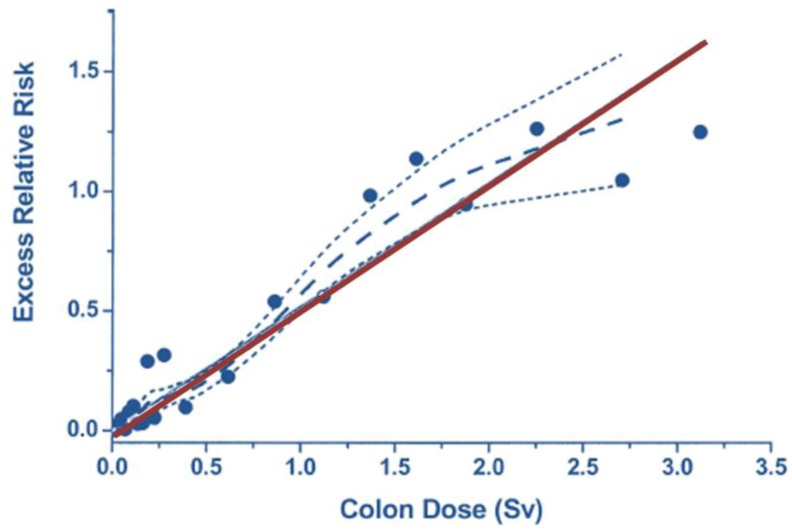
Excess relative risk of solid cancers derived from the Life Span Study cohort of A-bomb survivors over the dose range 0 to 2 Sv, to which a straight line is fitted. The solid straight line is the linear slope estimate, the points are dose category-specific ERR estimates, the dashed curve is a smoothed estimate derived from the points. The dotted curves indicate upper and lower one-standard-error bounds on the smoothed estimate (reproduced with permission from [8]).

**Figure 4:**
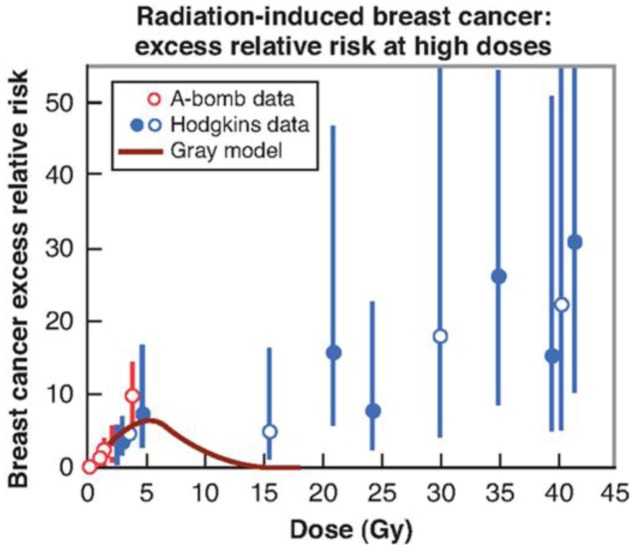
Excess relative risk for radiation-induced breast cancer. The low-dose data points are from A-bomb survivors (<4 Gy). The data points at high doses are from studies of SMN after radiotherapy of Hodgkin disease patient. The excess relative risk does not fall at high doses as predicted by the standard (Gray) model (reproduced with permission from [10]).

The risk of SMN following exposure to low and intermediate doses of radiation is well understood based on the data derived from A-bomb survivors. The data derived is limited to exposures of up to 2 Sv, and the dose-response curve is found to be a linear function of dose (Figure 3) [8]. Although this is useful to predict the risk of SMN at lower radiation doses, it may not be directly applicable in the context of radiotherapy, where limited volumes of tissue often deliberately receive doses of 70 Gy or higher, whereas a much larger volume receives a lower dose [22]. Also, the dose during the A-bomb was delivered as a single acute exposure of photons and neutrons to the whole body, unlike clinical radiotherapy. SMN risk at low doses (<100 mSv) has been derived assuming a linear-no-threshold model, indicating a finite probability of developing SMN even for the lowest doses of radiation exposure [9]. For doses >2 Sv, epidemiological data from patients undergoing radiotherapy indicates an increase in the risk of SMN with an increasing dose of radiation [4]. It was previously believed that the dose-response for radiation induced SMN followed a bell-shaped curve, where the risk of SMN decreased with an increase in the radiation dose due to the killing of cells (Gray model) [30]. But data from several epidemiological studies indicates that the SMN risk continues to increase even at large doses of up to 45 Gy (Figure 4) [10]. This has been explained due to the accelerated repopulation of cells occurring during and after fractionated high dose radiation [10]. The repopulation counteracts the cell killing effect at higher doses, and could explain the SMN risk observed at high doses after clinical radiotherapy. This new model which incorporates carcinogenic effects, cell killing effects, and cell repopulation effects of radiation could be used to predict and analyze the SMN risk at high radiation doses such as in IMRT. Available data also suggests that the fractionation of radiation dose does not necessarily decrease the risk of radiation induced SMN [4]. After a review of available animal and human data, Suit *et al.* concluded that the risk of SMN would be greatly reduced if normal tissue radiation exposures were maintained below 2 Gy during radiotherapy [4].

## 8. Radiation Technique

Radiotherapy techniques have evolved over the past 50 years from using superficial X-ray units to megavoltage linear accelerators. More recently, highly conformal radiation techniques, such as intensity modulated radiation therapy (IMRT) and charged particle therapy (proton radiation therapy), are being used more frequently. There is also an increasing use of imaging technologies such as cone beam CT for daily verification.

Use of older and earlier era treatment techniques has been shown to increase the risk of subsequent SMN [26]. More recently, there is a growing concern in the oncology community over the risk of SMN being induced by the increasing use of IMRT [19]. With the IMRT technique, patients are exposed to small volumes of high dose, and large volumes of normal tissue are exposed to a lower dose. This is in contrast to older 3D techniques, where large volumes of tissue were exposed to high doses of radiation. This would suggest that increasing the use of the IMRT may lead to a decrease in the future incidence of SMN, due to only small volumes of tissue being irradiated to a high dose. This may not be the case if one takes into account the effect of the scatter dose from the treatment-unit leakage. The leakage radiation emanating from the treatment unit is increased by a factor of 3 to 10 in IMRT compared to 3D techniques, and thus delivers a higher integral dose to the patient. Long-term outcomes data would be needed to draw firm conclusions about the effects of the IMRT technique on the risk of SMN. Use of proton beam therapy is believed to lower the predicted risk of SMN compared to photon therapy for some treatment sites [22,31], but long-term follow-up data will be needed to confirm these conclusions. Image Guided Radiation Therapy (IGRT) is increasingly being used in clinical radiotherapy. Use of IGRT for set-up verification during radiotherapy is known to contribute about 5–20% of the total dose to the organs outside the primary treatment field [32]. However, with the routine use of daily portal imaging or MV cone beam CT, exposures of up to 100 mGy per day are possible [22]. This could potentially increase the long-term risk of SMN, especially in younger patients. 

## 9. Quality of Radiation

The majority of the clinical experience in clinical radiotherapy has been with the use of photons, which are considered to have a low linear energy transfer (LET), as they cause sparse ionization along the DNA tract. More recently, particle therapy utilizing high energy protons and carbon ions are being used in the treatment of solid tumors (hadrontherapy). The relative biological effectiveness (RBE) of these particles varies as a function of LET, charge, and dose. Current evidence suggests that the basic mechanisms underlying carcinogenesis following high-LET radiation is similar to that of low-LET radiation [33]. However, due to the differences in the biological effects between high-LET radiation and X-rays, there are large uncertainties in predicting the SMN risk following heavy ion irradiation using epidemiological data from A-bomb survivors [33]. The SMN risk following exposure to leakage neutrons should be taken into account during the treatment with high energy photons and particle therapies. Neutrons are produced due to photonuclear interaction in the treatment hardware when photons >10 MV are used during radiation therapy [34]. The yield of photo-neutrons increases as the beam energy is increased. Neutrons are also produced in the treatment unit and in the patient during passive beam scanning technique in proton therapy, and by nuclear fragmentation in heavy ion therapy. These neutrons undergo collision interactions with protons resulting in secondary charged particles. Neutron radiation is known to be one the most effective in inducing late effects in the biological system, and thus has a high potential for induction of SMN [35]. Although survivors of Hiroshima were exposed to whole body neutrons, there are large uncertainties in the RBE values for neutron induced carcinogenesis [36]. This is due to the fact that, most of the carcinogenesis data is derived from low energy neutrons (<10 Mev), but neutrons in modern radiotherapy often have energies >100 Mev [36].

## 10. SMN after Treatment of Prostate Cancer

SMNs have been reported after radiation therapy for prostate cancer [37,38,39,40]. A recent retrospective study from Memorial Sloan-Kettering Cancer Hospital reported that the likelihoods of SMN after a 10-year follow-up following IMRT external beam radiation therapy (EBRT) and brachytherapy were 25% and 10%, respectively [37]. The most common SMNs included colorectal, bladder, skin, lymphoma and lung cancer. The increase in the SMN in the EBRT group was attributed to a statistically greater incidence of skin cancers from EBRT compared to brachytherapy treatment. This study is notable because of the long term follow-up of patients who were treated using the IMRT technique. A SEER analysis by Brenner *et al.* have reported a 34% increase in the risk of SMN in patients surviving more than ten years following radiation therapy, compared with those undergoing surgery [40]. However, many studies have failed to demonstrate a statistically significant association between SMN and radiation therapy in prostate cancer [41,42,43,44]. This could probably be due to the short follow-up time, reporting errors, and the use of tumor registry databases for risk calculation. Compared to the incidence, the mortality from SMN following radiation therapy is reported to be much lower at <5% [37]. In summary, radiation therapy has been implicated in the development of solid tumors in prostate cancer survivors both overall, and in areas that were exposed to radiation. The risk seems to increase with the follow-up time, but also seems to be lower with brachytherapy than with EBRT.

## 11. SMN after Treatment of Breast Cancer

Breast cancer is one of the most common cancers in women worldwide. Most of the time radiation is included as a part of breast cancer management. In one of the largest meta-analyses, which included 42,000 patients undergoing breast cancer treatment from 78 randomized trials, a 20% increase in the incidence of SMN, especially in contra-lateral breast cancer and lung cancer, was found in women undergoing radiation therapy compared to patients who received no additional radiation treatment [45]. Historical data suggests that the relative risk (RR) of SMN following breast radiation therapy is about 2, but the incidence in the modern era seems to be much lower due to advances in the planning technique and dosimetry [46,47,48]. SMN associated with breast cancer radiotherapy includes those of the lung, esophagus, contralateral breast, and sarcoma [49,50,51,52,53]. Factors associated with an increased incidence of SMN after breast radiotherapy include younger age, dose of radiation, and volume of normal tissue in the radiation field [47,48,53].

## 12. SMN after Treatment of Gynecological Malignancies

SMN has been reported following radiotherapy for endometrial and cervical cancer [17,54]. Chaturvedi *et al.* compiled a cohort of 104,760 cervical cancer patients using cancer registry data from Denmark, Finland, Norway, Sweden, and the United States, out of which 52,613 patients received radiation in the form of EBRT or intra-cavitary brachytherapy, or both. A 12% increase in the incidence of SMN including those of colon, anus/rectum, bladder, ovary, and genital sites were observed at a median follow-up of 12.2 years compared to the patients who did not receive radiation therapy [17]. Patients <50 years of age at diagnosis of cervical cancer were found to have a higher 40-year cumulative risk, than compared to women diagnosed after age 50. In the landmark Post-Operative Radiation Therapy in Endometrial Carcinoma (PORTEC)-1 trial, patients were randomly assigned to EBRT and no further treatment. About 22% of patients who received EBRT had SMN at 15 years, compared to 16% in patients who did not receive additional treatment. The most common SMN among EBRT patients was gastro-intestinal cancer (6.2%). This was nearly double the incidence compared to patients who received no additional treatment [54].

## 13. SMN after Treatment of Testicular Cancer

Until recently, the use of EBRT as adjuvant treatment after radical orchiectomy has been the standard of care for patients with early stage seminoma. Long-term survivors of testicular cancer have a statistically higher risk of SMN, even 35 years after radiation treatment, compared to the general population [55]. The estimated RR of SMN in these patients is about 1.4 to 2.8 [55]. However, the risk of SMN in testicular cancer has been much lower recently. This could be attributed to the decrease in the radiation field size and dose, the use of chemotherapy in place of radiation, and the adoption of surveillance/screening for early stage seminoma. There is a growing concern for those patients who receive continuous imaging surveillance as part of the observation process for early stage seminoma.

## 14. SMN after Treatment of Lymphoma

Many studies have reported an increase in the incidence of SMN in patients receiving radiation therapy for both Hodgkin lymphoma (HL) and non-Hodgkin lymphoma (NHL). The risk is especially concerning in patients with HL because most of the cases occur in young adults and are highly curable, and death from SMN can lead to a decrease in overall survival. For survivors of HL, there is an increased risk of multiple SMNs compared to the general population. These include breast, lung, colorectal, thyroid, sarcoma, and stomach cancers [56,57,58]. Similarly, NHL patients who were treated with radiation have an increased risk for SMNs in the form of both solid tumors and leukemia [59,60,61]. Combined modality treatments with cytotoxic drugs, a large irradiation field, younger age, and an increased radiation dose have all been identified as risk factors for SMN [62]. Historically, relatively large irradiation fields (mantle field) were used in the treatment of lymphoma. However, in order to decrease the risk of subsequent SMN, there has been a greater emphasis on utilizing lower doses of radiation and smaller treatment fields (involved field radiation therapy), especially in younger patients [63]. However, this risk seems to still exist even when lower doses of radiation have been used in pediatric patients [58].

## 15. SMN after Treatment of Pediatric Malignancies

Numerous studies have documented an increased risk of SMN after radiotherapy and chemotherapy in pediatric cancer patients compared to the general population [26,64,65,66,67]. The Childhood Cancer Survivor Study (CCSS) has provided valuable information on the incidence of SMN and late adverse effects after treatment of pediatric cancer (Table 2). CCSS includes 20,346 childhood cancer survivors diagnosed between 1970 and 1986. The cumulative incidence of all subsequent neoplasm after 30 years following diagnosis was 20.5% and was higher in patients who received radiation therapy [26]. Similarly, the British Childhood Cancer Survivor Study (BCCSS) reported a 13% incidence of SMN after a median follow-up of 24.3 years compared to the general population [68]. The use of radiation increased the RR of SMN including glioma and colorectal cancer. Similar to the findings in the BCCSS, Henderson *et al.* found an increased number of GI cancers in childhood cancer survivors as compared to the general population [69]. Eighty-seven percent of the childhood cancer survivors who later developed a GI SMN received radiotherapy for treatment of the primary cancer [69]. Another study from St. Jude Children’s Research Hospital evaluated over 1,600 patients with acute lymphoblastic leukemia, who received prophylactic cranial irradiation after primary chemotherapy. There was an increase in the incidence of low-grade gliomas, high-grade gliomas and meningiomas during the follow-up period of 20 years. The risk of brain tumors increased significantly with increasing radiation dose and in patients less than 6 years of age [70].

**Table 2:** Invasive second malignant neoplasms (SMNs) according to the latest Childhood Cancer Survivor Study (adapted from Reference [26] with author permission).

Invasive SMNs	Number	Median time to SMN occurrence in years, (range)
All invasive second malignancies	802	17.8 (5.0–35.2)
Leukemia	41	8.9 (5.0–31.1)
ALL	10	11.5 (6.1–26.5)
AML	21	7.4 (5.0–25.0)
CNS tumor	77	13.2 (6.0–32.7)
Glial	52	11.7 (6.0–25.5)
Medulloblastoma or PNET	6	11.6 (8.0–14.6)
Malignant Meningioma	11	22.9 (15.8–32.7)
Breast cancer	188	21.3 (6.7–33.5)
Melanoma	48	18.9 (5.6–35.2)
Thyroid cancer	128	18.6 (6.3–34.0)
Bone cancer	45	9.8 (5.3–26.7)
Osteosarcoma	35	9.3 (5.3–24.0)
Ewing sarcoma	4	9.3 (5.3–24.0)
Lymphoma	33	18.5 (6.9–31.5)
Hodgkin lymphoma	9	18.5 (7.2–29.1)
Non-Hodgkin lymphoma	21	21.6 (6.9–31.5)
Soft tissue sarcoma	73	15.2 (5.3–31.3)
Kidney cancer	20	19.6 (6.3–28.4)
Head and neck cancer	38	15.6 (5.3–30.9)
Small intestine and colorectal cancer	27	23.1 (7.0–29.4)
Lung and bronchus	11	20.3 (14.0–25.6)
Female genital cancers	23	19.5 (10.4–32.9)
Other cancers	50	21.0 (8.2–35.0)

According to the latest CCSS report, risk for developing subsequent SMN includes the female sex, younger age at diagnosis, radiation therapy, older treatment techniques, and primary diagnosis of HL [26]. Radiation therapy was the strongest independent risk factor for subsequent SMN on multivariate analysis [26]. There was an increased risk of all types of SMN including central nervous system tumors, soft tissue sarcomas, bone tumors, breast cancer, non-melanoma skin cancer and thyroid cancer after radiation therapy. The RR for SMN following radiotherapy was found to be 2.7. The CCSS study provides one of the best long term follow-up data on SMN following radiotherapy in pediatric patients.

## 16. Conclusion and Future Directions

Radiotherapy is a double edged sword. It has a well-established role in the curative treatment of various solid tumors. Unfortunately, radiation has the potential to induce cancer decades after the treatment. This is concerning as there is an increase in the number of long-term cancer survivors. There is an uncertainty in estimating the exact incidence of radiation induced SMN because of the confounding factors such as patient lifestyle and genetic susceptibility.

In the meantime, every effort should be made to minimize the influence of factors that could potentially increase the risk of SMN after radiotherapy. A lower total dose of radiation or non-radiation approach could be chosen for treatment whenever evidence supports the benefit without compromising tumor cure. The replacement of prophylactic cranial radiotherapy in children with leukemia by intrathecal chemotherapy is an example of this approach. The goal of radiation treatment planning should be to keep the normal tissue exposures to a minimum, more so in pediatric and younger patients. Daily image guidance should be used judiciously to minimize additional cumulative dose at the end of the treatment course. Novel treatment techniques such as the scanned beam proton radiation might decrease normal tissue exposure to leakage neutrons and it also might reduce SMN development.

Integrated research involving clinical studies, radiobiology, and physics is required for the estimation and reduction in the risk of radiation induced SMN. Prospective cohort studies for risk estimation should be designed (a) to include a large number of patients with sufficient statistical power (b) have a comparison group, where the same cancer was treated by means other than radiation, and (c) allow sufficiently long follow-up for radiation induced SMN to manifest. Radiobiological studies should be aimed at elucidating genetic mechanisms that might predispose individuals to develop SMN following radiotherapy, and to identify potential biomarkers in patients who are at risk for developing SMN. Efforts should also be made to develop mitigation strategies for patients at high risk for developing SMN following radiotherapy. Recent modeling studies have predicted a decrease in the risk of SMN following particle therapies compared to photon therapies. Long-term outcome data is awaited to firmly establish this to be the case.
